# Data mining and safety analysis of voriconazole in patients with a hematological malignant tumor based on the FAERS database: differences between children and adults

**DOI:** 10.3389/fphar.2025.1524702

**Published:** 2025-01-24

**Authors:** Hao Li, Manxue Jiang, Xiaona Pan, Lingti Kong

**Affiliations:** ^1^ Department of Pharmacy, The First Affiliated Hospital of Bengbu Medical University, Bengbu, China; ^2^ School of Pharmacy, Bengbu Medical University, Bengbu, China; ^3^ School of Fundamental Sciences, Bengbu Medical University, Bengbu, China; ^4^ Institute of Emergency and Critical Care Medicine, The First Affiliated Hospital of Bengbu Medical University, Bengbu, China

**Keywords:** data mining, voriconazole, hematological malignant tumor, FAERS, children, adults

## Abstract

**Objective:**

Voriconazole is a broad-spectrum antifungal agent. It is used widely for the prevention and treatment of invasive fungal infections in patients with a hematological malignancy, but studies on its safety in this population are scarce. We assessed the adverse drug events (ADEs) of voriconazole in this population based on the US Food and Drug Administration Adverse Event Reporting System (FAERS) database to improve understanding of the safety of voriconazole.

**Research design and methods:**

ADE reports for patients with a hematological malignant tumor using voriconazole between the first quarter of 2004 to the first quarter of 2024 were retrieved. Then, they were classified using the preferred terminology (PT) and system organ category (SOC) in the Medical Dictionary for Regulatory Activities. Data mining was done using reporting odds ratio (ROR), proportional reporting ratio (PRR), Bayesian confidence propagation neural network (BCPNN), and multi-item gamma Poisson shrinker (MGPS).

**Results:**

A total of 605 ADEs were included: 116 (19.17%) in children and 489 (80.83%) in adults. The types of SOC involved in children and adults were 22 and 24, respectively. The only positive SOC signal that satisfied all four algorithms simultaneously in children was “psychiatric disorders”, whereas in adults they were “endocrine disorders” and “hepatobiliary disorders”. At the PT level, the types involved in children and adults were 28 and 74, respectively. The highest ROR signal intensities were found for “hallucinations, mixed” in children and “toxic optic neuropathy” in adults. The median time of onset of the ADE in children and adults was 11 and 8.5 days, respectively.

**Conclusion:**

We used four algorithms (ROR, PRR, BCPNN, MGPS) to mine the signals of voriconazole in patients with a hematological malignant tumor, and compared the differences between children and adults. This study is important for targeting the monitoring, and could help to improve the safety of voriconazole.

## 1 Introduction

According to studies using cell lines involved in tumor transformation, the World Health Organization (WHO) classifies hematological malignant tumors into “myeloid tumours”, “lymphoid tumours”, “mast cell disorders”, and “histiocyte tumours” (Arber et al., 2016; Mertowska et al., 2023; Swerdlow et al., 2016). In patients suffering from a hematological malignancy, due to the fact that their immune system may be suppressed by the disease or treatment, these patients are more susceptible to invasive fungal infections (IFIs). In addition, patients with hematological malignant tumors often require the use of central venous catheters to provide medication, fluids, or nutrition, which also increases the risk of infection. Therefore, these patients are a high-risk group for IFIs and require special prevention and treatment strategies (Jenks et al., 2020; Pagano et al., 2011).

Voriconazole is a broad-spectrum antifungal agent belonging to the triazole class. It inhibits ergosterol biosynthesis in the membranes of fungal cells. Voriconazole is active against a wide range of fungi, including *Aspergillus* spp, *Candida* spp, and several others (Fernández Ávila et al., 2021; Purkins et al., 2003; Xie et al., 2023). Voriconazole is used as first-line treatment for invasive aspergillosis. It can also be used for prophylaxis in high-risk patients with an IFI. Voriconazole is active against a wide range of fungi, but it produces adverse effects, including hepatotoxicity, visual disturbances, and rash (Eiden et al., 2007; Yasu et al., 2022; Kim et al., 2017; Mihăilă, 2015; Wu et al., 2021; Xie et al., 2023). Studies have shown that adjustments to dosing regimens for voriconazole based on therapeutic drug monitoring are beneficial for promoting its safety and efficacy (Luong et al., 2016; Valle-T-Figueras et al., 2021).

There are significant differences in physiology and pharmacokinetics between children and adults,but the dosage for children is usually extrapolated from the adult dose (Leroux et al., 2021; Sienkiewicz-Oleszkiewicz et al., 2023; Wei et al., 2019). Children and adults may have different sensitivity and tolerance to drugs, leading to differences in the types and incidence of adverse drug events (ADEs) (Kearns et al., 2003; Pasternak et al., 2019). Therefore, grouping patients in these two age groups can more accurately evaluate the safety and efficacy of voriconazole in patients with hematological malignancies, providing more specific guidance for clinical medication.

The US Food and Drug Administration Adverse Event Reporting System (FAERS) is one of the major databases for the post-marketing surveillance of drugs (Cirmi et al., 2020; Feng et al., 2022). It is updated quarterly and publicly available for free download (Sakaeda et al., 2013). The database is used widely in pharmacovigilance studies to compensate for the limitations of the pre-marketing studies of drugs. FAERS plays an important part in updating drug inserts and releasing information on drug-safety alerts (Raisch et al., 2014).

Herein, we undertook data mining of ADEs with voriconazole in patients with a hematological malignancy using FAERS. We compared the differences between children and adults to provide information on the safety of voriconazole.

## 2 Materials and methods

### 2.1 Data sources

This study is based on data from FAERS from the first quarter of 2004 to the first quarter of 2024 (Zhai et al., 2019). FAERS consists of seven American Standard Code for Information Interchange data files: Demographic and Management Information, Adverse Drug Reaction Information, Patient Information, Drug Information, Date of Start and End of Treatment, Reporting Source Information, and Indication of Use/Diagnosis (Yu and Liu, 2024). The most recent FDA_DT with the same CASE ID, or a higher PRIMARY ID when the CASE ID and FDA_DT were identical, was selected to identify and remove duplicate reports (Huang et al., 2024; Zhou et al., 2022).

### 2.2 Data filtering

**FIGURE 1:**
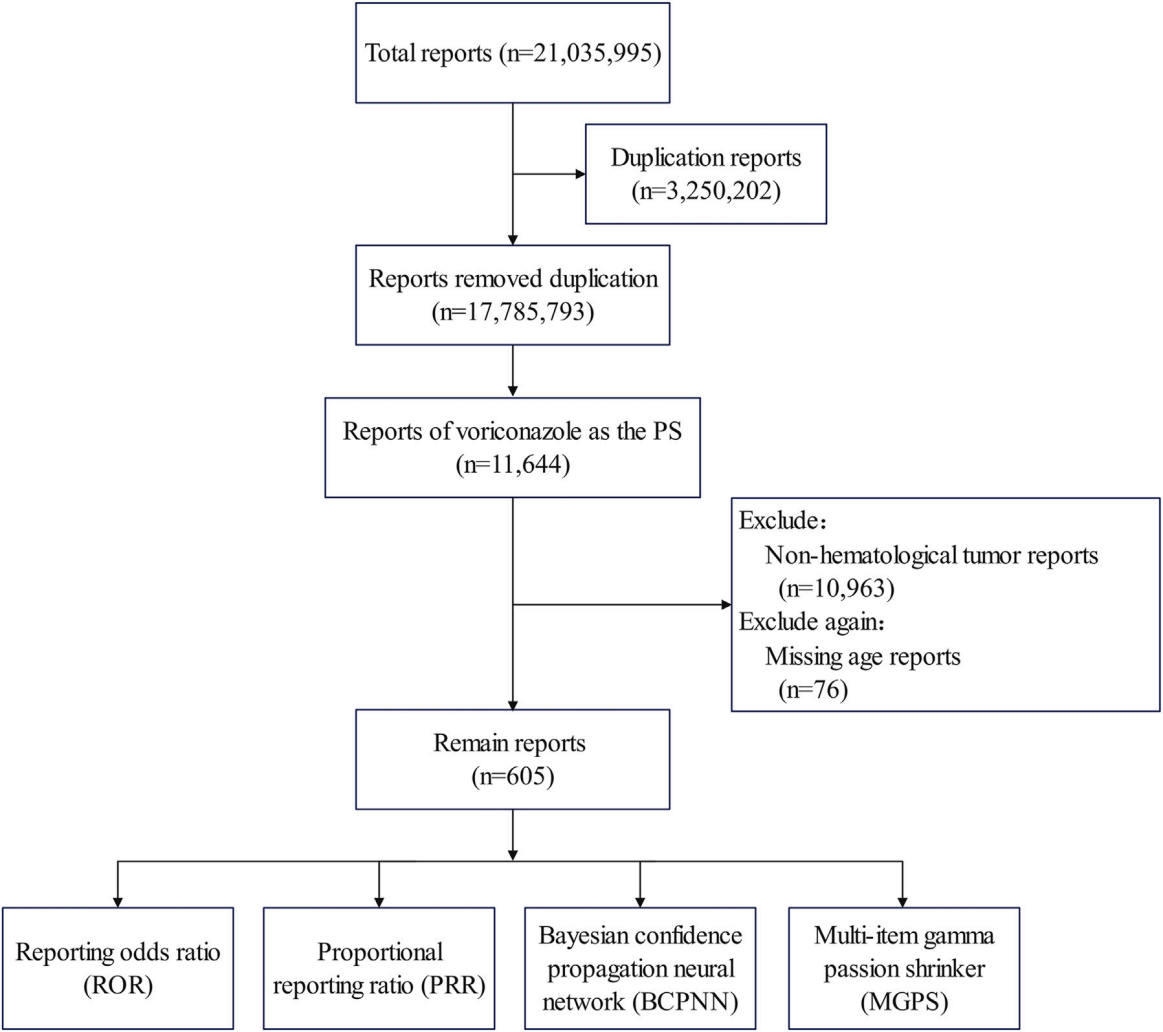
Flowchart showing data filtering.

The search was carried out using the drug names (voriconazole or Vfend) as the primary suspect in the ROLE field. Only patients identified as having a hematological malignant tumor were included. In addition, to compare the differences between adults and children, ADEs with missing ages were excluded (76 cases). The screening process for ADEs is shown in Figure 1.

ADEs were described and classified using the preferred terminology (PT) and the system organ category (SOC) in Medical Dictionary for Regulatory Activities v.26.0 (Romão et al., 2024).

### 2.3 Data mining

With respect to the safety evaluation of drugs, there are four commonly used signal-mining methods: reporting odds ratio (ROR), proportional reporting ratio (PRR), Bayesian confidence propagation neural network (BCPNN), and multi-item gamma Poisson shrinker (MGPS) (Jiang et al., 2024; Liu et al., 2024; Xiong et al., 2024). The calculation and judgment criteria for these four signal mining methods are shown in Supplementary Table S1. In this study, to remove bias, only those that met all the criteria of the four algorithms were considered to be positive signals (Zhang et al., 2024).

We also assessed the time-to-onset of the ADE, which was defined as the interval between the onset date (EVENT_DT) and start date (START_DT).

### 2.4 Statistical analyses

Data were analyzed using SPSS 26.0 (IBM, Armonk, NY, United States). Descriptive statistics were used. Variables are presented as numbers and percentages. R 4.3.1 (R Institute for Statistical Computing, Vienna, Austria) was employed for data visualization.

## 3 Results

### 3.1 Descriptive characteristics

**FIGURE 2:**
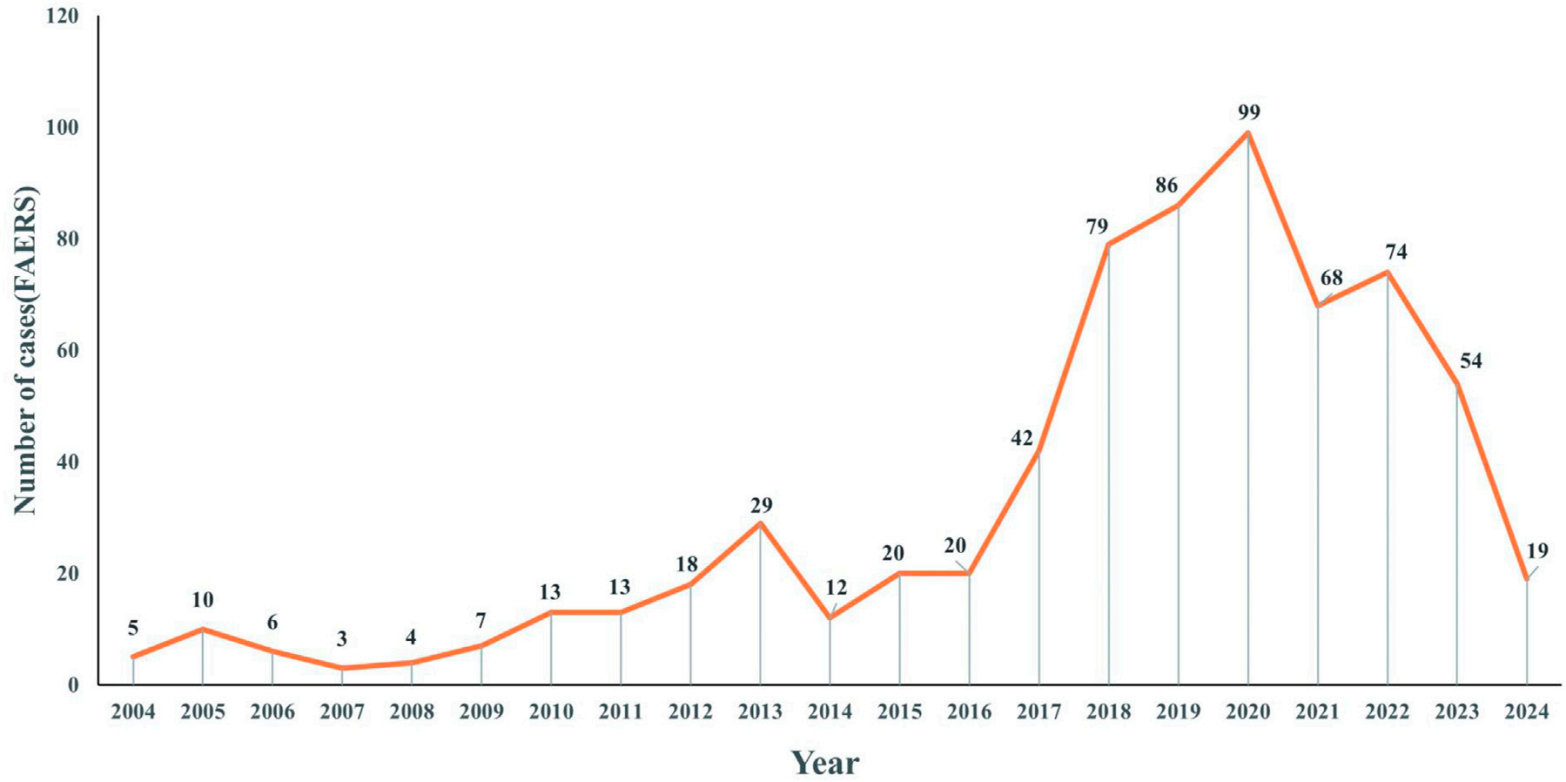
Distribution of reported trends in the use of voriconazole ADEs in patients with a hematological malignant tumor from Q1 2004 to Q1 2024.

Figure 2 presents the annual distribution of ADEs related to voriconazole use in patients with a hematological malignancy: there was a general upward trend until 2020.

The number of children and adults was 116 (19.17%) and 489 (80.83%), respectively (Table 1). The study cohort comprised 214 females (35.37%), 372 males (61.49%), and 19 unspecified cases (3.14%). The country that provided the most ADEs was the USA, followed by France. The main reporter type was “professionals” (physician, pharmacist, health-professional), accounting for 88.26%, which greatly improved the reliability of ADE information.

**TABLE 1:** Demographic characteristics of ADEs reported in FAERS (first quarter of 2004 to first quarter of 2024) with voriconazole use as the main suspected drug in patients with a hematological malignancy.

Characteristic	Children	Adults
Sex		
Male	68 (58.62%)	304 (62.17%)
Female	47 (40.52%)	167 (34.15%)
Data missing	1 (0.86%)	18 (3.68%)
ADEs	116 (100%)	489 (100%)
Weight (kg)		
<50	19 (16.38%)	8 (1.64%)
50–100 g	4 (3.45%)	9 (1.84%)
>100	0 (0.00%)	104 (21.27%)
Data missing	93 (80.17%)	368 (75.26%)
Reporter country		
United States	16 (13.79%)	161 (32.92%)
France	8 (6.90%)	83 (16.97%)
German	1 (0.86%)	38 (7.77%)
Japan	13 (11.21%)	30 (6.13%)
Italy	5 (4.31%)	23 (4.70%)
Polish	4 (3.45%)	16 (3.27%)
China	7 (6.03%)	16 (3.27%)
Netherlands	13 (11.21%)	13 (2.66%)
Other	47 (40.52%)	103 (21.06%)
Data missing	2 (1.72%)	6 (1.23%)
Reporter type		
Physician	26 (22.41%)	162 (33.13%)
Pharmacist	7 (6.03%)	18 (3.68%)
Health-professional	76 (65.52%)	245 (50.10%)
Consumer	4 (3.45%)	57 (11.66%)
Data missing	3 (2.59%)	7 (1.43%)

Abbreviation: ADEs, adverse drug events.

### 3.2 Signal detects at the SOC level

At the SOC level (Tables 2, 3), the number of ADEs caused by voriconazole in children and adults was 22 and 24, respectively. The only positive signal that satisfied all four algorithms simultaneously in children was “psychiatric disorders”, whereas in adults it was “endocrine disorders” and “hepatobiliary disorders”.

**TABLE 2:** Frequency and signal intensity of ADEs in children at the level of system organ classifications (SOC).

SOCs	Frequency	ROR (95%Cl)	PRR (χ^2^)	EBGM (EBGM05)	IC (IC025)
Psychiatric disorders	31	3.46 (2.4–4.98)	3.32 (50.05)	3.27 (2.41)	1.71 (0.04)
Skin and subcutaneous tissue disorders	47	3.08 (2.28–4.16)	2.91 (59.34)	2.87 (2.23)	1.52 (−0.15)
General disorders and administration site conditions	124	2.45 (2.01–2.99)	2.14 (82.35)	2.12 (1.79)	1.08 (-0.59)
Product issues	1	1.98 (0.27–14.26)	1.98 (0.48)	1.96 (0.38)	0.97 (-0.73)
Ear and labyrinth disorders	1	1.95 (0.27–14.06)	1.95 (0.46)	1.94 (0.37)	0.95 (-0.75)
Injury, poisoning and procedural complications	45	1.67 (1.23–2.26)	1.61 (10.9)	1.61 (1.24)	0.68 (-0.99)
Hepatobiliary disorders	22	1.35 (0.88–2.07)	1.34 (1.89)	1.33 (0.93)	0.41 (-1.26)
Renal and urinary disorders	15	1.23 (0.74–2.07)	1.23 (0.64)	1.23 (0.8)	0.29 (-1.38)
Infections and infestations	81	1.12 (0.88–1.42)	1.1 (0.89)	1.1 (0.91)	0.14 (-1.53)
Musculoskeletal and connective tissue disorders	12	1.04 (0.59–1.85)	1.04 (0.02)	1.04 (0.64)	0.05 (-1.62)
Gastrointestinal disorders	42	0.87 (0.63–1.19)	0.88 (0.81)	0.88 (0.67)	−0.19 (−1.86)
Cardiac disorders	10	0.82 (0.44–1.54)	0.83 (0.37)	0.83 (0.49)	−0.27 (−1.94)
Nervous system disorders	49	0.75 (0.56–1)	0.77 (3.76)	0.77 (0.6)	−0.37 (−2.05)
Investigations	34	0.67 (0.47–0.95)	0.69 (5.15)	0.69 (0.52)	−0.53 (−2.2)
Endocrine disorders	2	0.66 (0.16–2.64)	0.66 (0.35)	0.66 (0.21)	−0.6 (−2.27)
Respiratory, thoracic and mediastinal Disorders	13	0.51 (0.29–0.88)	0.52 (6.03)	0.52 (0.33)	−0.94 (−2.61)
Metabolism and nutrition disorders	10	0.48 (0.25–0.89)	0.49 (5.63)	0.49 (0.29)	−1.04 (−2.71)
Blood and lymphatic system disorders	25	0.37 (0.25–0.55)	0.39 (26.16)	0.4 (0.28)	−1.34 (−3.01)
Eye disorders	2	0.33 (0.08–1.33)	0.33 (2.69)	0.33 (0.1)	−1.58 (−3.25)
Vascular disorders	3	0.2 (0.07–0.63)	0.21 (9.37)	0.21 (0.08)	−2.27 (−3.94)
Immune system disorders	3	0.19 (0.06–0.58)	0.19 (10.7)	0.19 (0.07)	−2.39 (−4.06)
Neoplasms benign, malignant and unspecified (Incl Cysts And Polyps)	2	0.1 (0.02–0.38)	0.1 (16.99)	0.1 (0.03)	−3.33 (−5)

Abbreviations: SOCs, system organ classes; ROR, reporting odds ratio; PRR, proportional reporting ratio; EBGM, empirical bayes geometric mean; IC, information component; 95% CI, 95% confidence interval; χ2: Chi-squared.

**TABLE 3:** Frequency and signal intensity of ADEs in adults at the level of system organ classifications (SOC).

SOCs	Frequency	ROR (95% Cl)	PRR (χ^2^)	EBGM (EBGM05)	IC (IC025)
Endocrine disorders	18	5.82 (3.65–9.28)	5.78 (70.69)	5.74 (3.89)	2.52 (0.85)
Hepatobiliary disorders	77	3.46 (2.75–4.35)	3.36 (128.72)	3.35 (2.77)	1.74 (0.08)
Eye disorders	57	2.47 (1.9–3.21)	2.42 (48.14)	2.42 (1.94)	1.27 (−0.39)
Infections and infestations	365	1.89 (1.68–2.11)	1.72 (123.35)	1.72 (1.56)	0.78 (−0.89)
Psychiatric disorders	51	1.49 (1.13–1.97)	1.47 (7.92)	1.47 (1.17)	0.56 (−1.11)
General disorders and administration site conditions	374	1.38 (1.23–1.54)	1.31 (31.34)	1.31 (1.19)	0.38 (−1.28)
Cardiac disorders	72	1.13 (0.9–1.44)	1.13 (1.1)	1.13 (0.93)	0.18 (−1.49)
Nervous system disorders	144	1.07 (0.9–1.27)	1.07 (0.65)	1.07 (0.93)	0.09 (−1.57)
Injury, poisoning and procedural complications	131	1.03 (0.87–1.23)	1.03 (0.13)	1.03 (0.89)	0.04 (−1.62)
Investigations	179	1.01 (0.87–1.18)	1.01 (0.03)	1.01 (0.89)	0.02 (−1.65)
Renal and urinary disorders	35	0.86 (0.62–1.2)	0.86 (0.76)	0.86 (0.65)	−0.21 (−1.88)
Blood and lymphatic system disorders	104	0.81 (0.67–0.99)	0.82 (4.25)	0.82 (0.7)	−0.28 (−1.95)
Respiratory, thoracic and mediastinal disorders	84	0.78 (0.63–0.97)	0.79 (4.98)	0.79 (0.66)	−0.34 (−2.01)
Pregnancy, puerperium and perinatal conditions	1	0.78 (0.11–5.57)	0.78 (0.06)	0.78 (0.15)	−0.35 (−2.02)
Congenital, familial and genetic disorders	2	0.76 (0.19–3.04)	0.76 (0.15)	0.76 (0.24)	−0.4 (−2.07)
Immune system disorders	19	0.75 (0.48–1.17)	0.75 (1.61)	0.75 (0.51)	−0.42 (−2.08)
Vascular disorders	33	0.69 (0.49–0.97)	0.7 (4.5)	0.7 (0.52)	−0.52 (−2.19)
Reproductive system and breast disorders	3	0.64 (0.2–1.97)	0.64 (0.62)	0.64 (0.25)	−0.65 (−2.32)
Skin and subcutaneous tissue disorders	57	0.63 (0.48–0.82)	0.64 (11.91)	0.64 (0.52)	−0.64 (−2.31)
Metabolism and nutrition disorders	33	0.62 (0.44–0.88)	0.63 (7.42)	0.63 (0.47)	−0.67 (−2.34)
Neoplasms benign, malignant and unspecified (Incl Cysts and Polyps)	47	0.45 (0.33–0.6)	0.46 (31.53)	0.46 (0.36)	−1.12 (−2.79)
Gastrointestinal disorders	49	0.27 (0.21–0.36)	0.29 (92.49)	0.29 (0.23)	−1.78 (−3.45)
Musculoskeletal and connective tissue disorders	17	0.22 (0.14–0.36)	0.23 (45.21)	0.23 (0.16)	−2.11 (−3.78)
Surgical and medical procedures	2	0.11 (0.03–0.45)	0.11 (14.08)	0.11 (0.04)	−3.15 (−4.81)

Abbreviations: SOCs, system organ classes; ROR, reporting odds ratio; PRR, proportional reporting ratio; EBGM, empirical bayes geometric mean; IC, information component; 95% CI, 95% confidence interval; χ2, Chi-squared.

**FIGURE 3:**
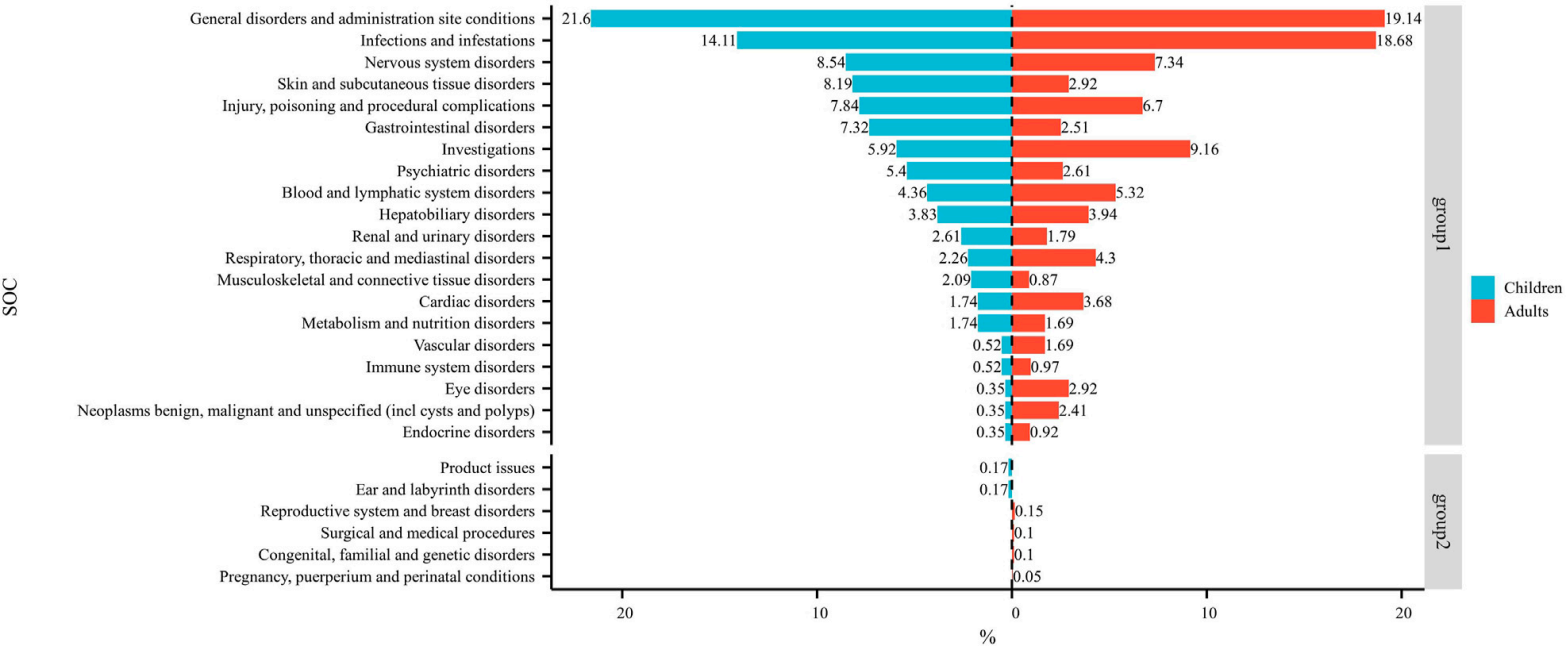
Dual-valued histograms plotted to depict the difference in SOCs between children and adults. *Note:* left: children; right: adult.

Figure 3 shows the frequency of each SOC in children and adults as a percentage of the total SOC. The SOCs shown in group 1 were present in children and adults. The SOCs in group 2 were present in children or adults.

### 3.3 Signal detects at the PT level

Table 4 shows all positive PTs of voriconazole in the child group (ranked by ROR), totaling 28 types involving 13 SOCs. The top-five PTs were “hallucinations, mixed” (ROR: 363.94), “labelled drug-drug interaction medication error” (ROR: 170.43), “photosensitivity reaction” (ROR: 116.47), “steatohepatitis” (ROR: 116.25), and “drug level below therapeutic” (ROR: 109.37).

**TABLE 4:** Frequency and signal intensity of ADEs in children at the level of preferred terms (PTs).

PTs	SOC	Frequency	ROR (95% CI)	PRR (χ^2^)	EBGM (EBGM05)	IC (IC025)
Hallucinations, mixed	Psychiatric disorders	5	363.94 (70.46–1879.79)	360.78 (512.59)	103.79 (26.27)	6.7 (4.74)
Labelled drug-drug interaction medication error	Injury, poisoning and procedural complications	7	170.43 (57.1–508.72)	168.36 (537.59)	78.24 (31.34)	6.29 (4.45)
Photosensitivity reaction	Skin and subcutaneous tissue disorders	21	116.47 (65.45–207.26)	112.24 (1,303.1)	63.57 (39.25)	5.99 (4.27)
Steatohepatitis	Hepatobiliary disorders	4	116.25 (31.14–434.05)	115.45 (252.16)	64.58 (21.45)	6.01 (4.11)
Drug level below therapeutic	Investigations	6	109.37 (37.83–316.22)	108.23 (364.32)	62.28 (25.62)	5.96 (4.13)
Cheilitis	Gastrointestinal disorders	14	76.67 (39.99–147)	74.83 (671.9)	49.62 (28.78)	5.63 (3.9)
Photodermatosis	Skin and subcutaneous tissue disorders	3	72.53 (18.09–290.72)	72.16 (140.35)	48.44 (15.16)	5.6 (3.69)
Hallucination, auditory	Psychiatric disorders	4	48.43 (15.57–150.63)	48.1 (138.4)	36.33 (14.06)	5.18 (3.37)
Musculoskeletal pain	Musculoskeletal and connective tissue disorders	6	36.45 (14.84–89.51)	36.08 (163.77)	29.06 (13.7)	4.86 (3.11)
Hallucination, visual	Psychiatric disorders	5	30.32 (11.53–79.75)	30.06 (116.31)	25.05 (11.15)	4.65 (2.9)
Hypercalcaemia	Metabolism and nutrition disorders	5	29.11 (11.1–76.3)	28.86 (112.11)	24.22 (10.81)	4.6 (2.85)
Inflammation	General disorders and administration site conditions	7	25.55 (11.4–57.29)	25.25 (138.85)	21.64 (11.01)	4.44 (2.71)
Disorientation	Psychiatric disorders	6	19.01 (8.09–44.69)	18.82 (89.63)	16.77 (8.2)	4.07 (2.35)
Rash macular	Skin and subcutaneous tissue disorders	3	18.92 (5.66–63.18)	18.82 (44.8)	16.77 (6.11)	4.07 (2.31)
Neuralgia	Nervous system disorders	7	17.93 (8.14–39.48)	17.72 (98.45)	15.89 (8.21)	3.99 (2.28)
Drug interaction	General disorders and administration site conditions	43	13.84 (10.01–19.12)	12.87 (435.1)	11.9 (9.08)	3.57 (1.9)
Hypoaesthesia	Nervous system disorders	6	10.93 (4.75–25.15)	10.82 (49.81)	10.14 (5.05)	3.34 (1.64)
Therapeutic response decreased	General disorders and administration site conditions	4	10.56 (3.81–29.24)	10.5 (32.06)	9.85 (4.2)	3.3 (1.59)
Drug level increased	Investigations	5	7.73 (3.13–19.09)	7.68 (27.6)	7.34 (3.45)	2.88 (1.18)
Agranulocytosis	Blood and lymphatic system disorders	4	6.67 (2.44–18.25)	6.64 (18.32)	6.39 (2.75)	2.68 (0.98)
Drug-induced liver injury	Hepatobiliary disorders	4	6.18 (2.26–16.86)	6.14 (16.53)	5.93 (2.56)	2.57 (0.87)
Condition aggravated	General disorders and administration site conditions	8	6.12 (3–12.47)	6.04 (32.4)	5.84 (3.22)	2.55 (0.86)
Arthralgia	Musculoskeletal and connective tissue disorders	4	5.38 (1.97–14.63)	5.34 (13.64)	5.19 (2.25)	2.38 (0.68)
Treatment failure	General disorders and administration site conditions	5	5.34 (2.18–13.09)	5.31 (16.88)	5.15 (2.43)	2.37 (0.68)
Respiratory distress	Respiratory, thoracic and mediastinal disorders	7	5.13 (2.4–10.94)	5.08 (22.19)	4.94 (2.62)	2.3 (0.62)
Confusional state	Psychiatric disorders	6	4.82 (2.13–10.93)	4.78 (17.42)	4.66 (2.35)	2.22 (0.54)
Drug ineffective	General disorders and administration site conditions	21	4.78 (3.07–7.44)	4.64 (58.59)	4.53 (3.13)	2.18 (0.5)
Septic shock	Infections and infestations	8	3.85 (1.9–7.81)	3.81 (16.22)	3.74 (2.07)	1.9 (0.22)

Abbreviations: PTs, preferred terms; SOC, system organ class; ROR, reporting odds ratio; PRR, proportional reporting ratio; EBGM, empirical bayes geometric mean; IC, information component; 95% CI, 95% confidence interval; χ2, Chi-squared.

Table 5 shows all positive PTs of voriconazole in the adult group (ranked by ROR), totaling 74 types involving 17 SOCs. The top-five PTs were “toxic optic neuropathy” (ROR: 2722.79), “drug level below therapeutic” (ROR: 348.91), “vascular access site infection” (ROR: 316.94), “peptic ulcer haemorrhage” (ROR: 308.45), and “drug level decreased” (ROR: 175.69).

**TABLE 5:** Frequency and signal intensity of ADEs in adults at the level of preferred terms (PTs).

PTs	SOC	Frequency	ROR (95% CI)	PRR (χ^2^)	EBGM (EBGM05)	IC (IC025)
Toxic optic neuropathy	Eye disorders	11	2,722.79 (759.01–9,767.45)	2,707.47 (6,377.23)	580.96 (199.5)	9.18 (7.36)
Drug level below therapeutic	Investigations	8	348.91 (150.4–809.42)	347.48 (1879.47)	236.61 (117.01)	7.89 (6.13)
Vascular access site infection	Infections and infestations	3	316.94 (81.9–1,226.57)	316.46 (660.37)	221.82 (71.49)	7.79 (5.91)
Peptic ulcer haemorrhage	Gastrointestinal disorders	5	308.45 (108.56–876.39)	307.67 (1,078.85)	217.47 (90.77)	7.76 (5.96)
Drug level decreased	Investigations	9	175.69 (84.84–363.82)	174.88 (1,258.09)	141.59 (77)	7.15 (5.43)
Endophthalmitis	Infections and infestations	6	113.95 (48.19–269.45)	113.6 (580.38)	98.59 (47.98)	6.62 (4.9)
Intentional overdose	Injury, poisoning and procedural complications	4	75.89 (27.09–212.56)	75.73 (267.55)	68.78 (29.05)	6.1 (4.38)
Superinfection bacterial	Infections and infestations	4	62.97 (22.67–174.94)	62.84 (224.35)	57.99 (24.66)	5.86 (4.14)
Hallucination, visual	Psychiatric disorders	9	49.45 (25.15–97.24)	49.23 (398.69)	46.21 (26.24)	5.53 (3.85)
Eastern cooperative oncology group performance status worsened	Investigations	3	46.22 (14.38–148.51)	46.15 (124.72)	43.49 (16.38)	5.44 (3.73)
Drug interaction	General disorders and administration site conditions	92	34.04 (27.49–42.15)	32.49 (2,693.48)	31.16 (26.06)	4.96 (3.29)
Drug level increased	Investigations	14	33.05 (19.31–56.55)	32.82 (413.59)	31.46 (20.07)	4.98 (3.3)
Contraindicated product administered	Injury, poisoning and procedural complications	5	32.75 (13.36–80.31)	32.67 (147.02)	31.33 (14.79)	4.97 (3.28)
Neurological decompensation	Nervous system disorders	7	32.62 (15.28–69.63)	32.51 (204.78)	31.18 (16.53)	4.96 (3.28)
Torsade de pointes	Cardiac disorders	6	30.65 (13.53–69.43)	30.55 (164.72)	29.38 (14.82)	4.88 (3.19)
Nephrotic syndrome	Renal and urinary disorders	7	29.14 (13.67–62.09)	29.04 (182.36)	27.98 (14.86)	4.81 (3.13)
Photosensitivity reaction	Skin and subcutaneous tissue disorders	9	28.28 (14.51–55.12)	28.16 (227.13)	27.16 (15.54)	4.76 (3.09)
Intervertebral discitis	Infections and infestations	4	26.66 (9.82–72.36)	26.61 (95.16)	25.72 (11.15)	4.68 (3)
Brain abscess	Infections and infestations	4	23.12 (8.54–62.61)	23.07 (81.92)	22.41 (9.74)	4.49 (2.8)
Central nervous system lesion	Nervous system disorders	6	22.79 (10.1–51.41)	22.72 (120.88)	22.07 (11.17)	4.46 (2.78)
Psychotic disorder	Psychiatric disorders	5	21.64 (8.88–52.72)	21.59 (95.41)	21.01 (9.97)	4.39 (2.71)
Pathogen resistance	Infections and infestations	6	21.16 (9.39–47.69)	21.1 (111.69)	20.54 (10.4)	4.36 (2.68)
Generalised tonic-clonic seizure	Nervous system disorders	8	20.81 (10.29–42.07)	20.73 (146.13)	20.19 (11.2)	4.34 (2.66)
Bacterial test positive	Investigations	3	20.17 (6.4–63.55)	20.14 (53.12)	19.63 (7.51)	4.29 (2.61)
Prescribed overdose	Injury, poisoning and procedural complications	3	19.81 (6.29–62.4)	19.78 (52.1)	19.29 (7.38)	4.27 (2.58)
Hallucination	Psychiatric disorders	17	19.41 (11.97–31.49)	19.25 (286.84)	18.79 (12.54)	4.23 (2.56)
Cholecystitis acute	Hepatobiliary disorders	6	16.83 (7.48–37.85)	16.78 (87.09)	16.43 (8.34)	4.04 (2.36)
Neurological symptom	Nervous system disorders	5	15.42 (6.35–37.43)	15.38 (65.88)	15.09 (7.19)	3.92 (2.24)
Hypertransaminasaemia	Hepatobiliary disorders	5	15.17 (6.25–36.81)	15.13 (64.67)	14.85 (7.07)	3.89 (2.22)
Nephropathy	Renal and urinary disorders	3	14.89 (4.74–46.73)	14.87 (38.04)	14.59 (5.6)	3.87 (2.18)
Hemiparesis	Nervous system disorders	7	13.51 (6.39–28.56)	13.46 (79.32)	13.24 (7.07)	3.73 (2.05)
Spinal cord compression	Nervous system disorders	4	13.45 (5–36.19)	13.43 (45.19)	13.2 (5.77)	3.72 (2.04)
Treatment failure	General disorders and administration site conditions	17	13.03 (8.05–21.09)	12.93 (183.99)	12.72 (8.5)	3.67 (2)
Nail disorder	Skin and subcutaneous tissue disorders	3	12.82 (4.09–40.18)	12.8 (32.1)	12.6 (4.85)	3.66 (1.97)
Ventricular hypokinesia	Cardiac disorders	3	12.82 (4.09–40.18)	12.8 (32.1)	12.6 (4.85)	3.66 (1.97)
Necrosis	General disorders and administration site conditions	3	12.26 (3.91–38.39)	12.24 (30.46)	12.06 (4.64)	3.59 (1.91)
Acute hepatic failure	Hepatobiliary disorders	6	11.94 (5.32–26.79)	11.91 (59.03)	11.74 (5.97)	3.55 (1.88)
Cardiovascular disorder	Cardiac disorders	5	11.86 (4.9–28.73)	11.83 (48.82)	11.66 (5.56)	3.54 (1.87)
Brain oedema	Nervous system disorders	5	11.71 (4.84–28.36)	11.68 (48.1)	11.52 (5.49)	3.53 (1.85)
Oral disorder	Gastrointestinal disorders	4	11.29 (4.2–30.35)	11.27 (36.89)	11.12 (4.86)	3.47 (1.8)
Neutrophil count increased	Investigations	4	10.27 (3.83–27.59)	10.26 (32.96)	10.13 (4.43)	3.34 (1.66)
Ventricular extrasystoles	Cardiac disorders	4	10.1 (3.76–27.12)	10.08 (32.28)	9.96 (4.36)	3.32 (1.64)
Inappropriate antidiuretic hormone secretion	Endocrine disorders	4	9.7 (3.61–26.04)	9.68 (30.75)	9.57 (4.19)	3.26 (1.58)
Herpes simplex	Infections and infestations	4	9.67 (3.6–25.96)	9.65 (30.63)	9.54 (4.18)	3.25 (1.58)
Hepatic cytolysis	Hepatobiliary disorders	7	8.94 (4.24–18.86)	8.91 (48.6)	8.82 (4.72)	3.14 (1.47)
Eyelid oedema	Eye disorders	3	8.87 (2.84–27.72)	8.86 (20.68)	8.77 (3.38)	3.13 (1.46)
Pulmonary mass	Respiratory, thoracic and mediastinal disorders	6	8.68 (3.88–19.43)	8.65 (40.16)	8.56 (4.36)	3.1 (1.43)
Ileus paralytic	Gastrointestinal disorders	4	8.55 (3.19–22.94)	8.54 (26.32)	8.45 (3.7)	3.08 (1.4)
Drug ineffective for unapproved indication	General disorders and administration site conditions	5	8.47 (3.5–20.47)	8.45 (32.47)	8.36 (4)	3.06 (1.39)
Hepatotoxicity	Hepatobiliary disorders	10	8.13 (4.35–15.19)	8.1 (61.56)	8.02 (4.75)	3 (1.33)
Ventricular fibrillation	Cardiac disorders	3	7.92 (2.54–24.73)	7.91 (17.92)	7.84 (3.02)	2.97 (1.29)
Haemoptysis	Respiratory, thoracic and mediastinal disorders	8	7.62 (3.79–15.31)	7.59 (45.35)	7.53 (4.2)	2.91 (1.24)
Electrocardiogram qt prolonged	Investigations	11	7.55 (4.16–13.7)	7.51 (61.54)	7.45 (4.52)	2.9 (1.23)
Cholestasis	Hepatobiliary disorders	7	7.49 (3.55–15.8)	7.47 (38.85)	7.4 (3.97)	2.89 (1.22)
Hypothyroidism	Endocrine disorders	6	7.48 (3.34–16.73)	7.46 (33.23)	7.39 (3.77)	2.89 (1.22)
Pulmonary haemorrhage	Respiratory, thoracic and mediastinal disorders	4	7.27 (2.71–19.48)	7.26 (21.38)	7.2 (3.15)	2.85 (1.17)
Blood alkaline phosphatase increased	Investigations	9	7.08 (3.66–13.66)	7.05 (46.29)	6.99 (4.03)	2.81 (1.14)
Ventricular tachycardia	Cardiac disorders	4	7.01 (2.62–18.79)	7 (20.38)	6.94 (3.04)	2.8 (1.12)
Respiratory disorder	Respiratory, thoracic and mediastinal disorders	7	6.92 (3.28–14.59)	6.9 (35.01)	6.85 (3.67)	2.78 (1.1)
Nephropathy toxic	Renal and urinary disorders	3	6.89 (2.21–21.49)	6.88 (14.94)	6.83 (2.63)	2.77 (1.1)
Drug ineffective	General disorders and administration site conditions	94	6.85 (5.56–8.43)	6.57 (443.05)	6.52 (5.48)	2.7 (1.04)
Skin infection	Infections and infestations	4	6.8 (2.54–18.22)	6.79 (19.57)	6.74 (2.95)	2.75 (1.08)
Guillain-barre syndrome	Nervous system disorders	3	6.8 (2.18–21.22)	6.8 (14.69)	6.74 (2.6)	2.75 (1.08)
Hepatocellular injury	Hepatobiliary disorders	6	6.3 (2.82–14.09)	6.28 (26.45)	6.24 (3.18)	2.64 (0.97)
Immunosuppression	Immune system disorders	4	6.2 (2.32–16.61)	6.19 (17.28)	6.15 (2.7)	2.62 (0.95)
Metabolic acidosis	Metabolism and nutrition disorders	4	6.1 (2.28–16.34)	6.09 (16.88)	6.05 (2.65)	2.6 (0.92)
Skin lesion	Skin and subcutaneous tissue disorders	8	5.72 (2.85–11.49)	5.7 (30.8)	5.67 (3.16)	2.5 (0.83)
Drug-induced liver injury	Hepatobiliary disorders	4	5.66 (2.11–15.14)	5.65 (15.19)	5.61 (2.46)	2.49 (0.82)
Left ventricular dysfunction	Cardiac disorders	3	5.57 (1.79–17.37)	5.57 (11.16)	5.53 (2.14)	2.47 (0.79)
Hepatitis	Hepatobiliary disorders	5	5.46 (2.26–13.19)	5.45 (18.06)	5.42 (2.59)	2.44 (0.77)
Product use in unapproved indication	Injury, poisoning and procedural complications	31	5.26 (3.69–7.51)	5.2 (104.61)	5.17 (3.83)	2.37 (0.7)
Thrombotic microangiopathy	Blood and lymphatic system disorders	4	5.09 (1.9–13.63)	5.08 (13.04)	5.06 (2.22)	2.34 (0.67)
Condition aggravated	General disorders and administration site conditions	16	4.79 (2.92–7.84)	4.75 (47.21)	4.73 (3.13)	2.24 (0.57)
Graft versus host disease	Immune system disorders	5	4.51 (1.87–10.87)	4.5 (13.52)	4.48 (2.14)	2.16 (0.49)

Abbreviations: PTs, preferred terms; SOC, system organ class; ROR, reporting odds ratio; PRR, proportional reporting ratio; EBGM, empirical bayes geometric mean; IC, information component; 95% CI, 95% confidence interval; χ2, Chi-squared.

**FIGURE 4:**
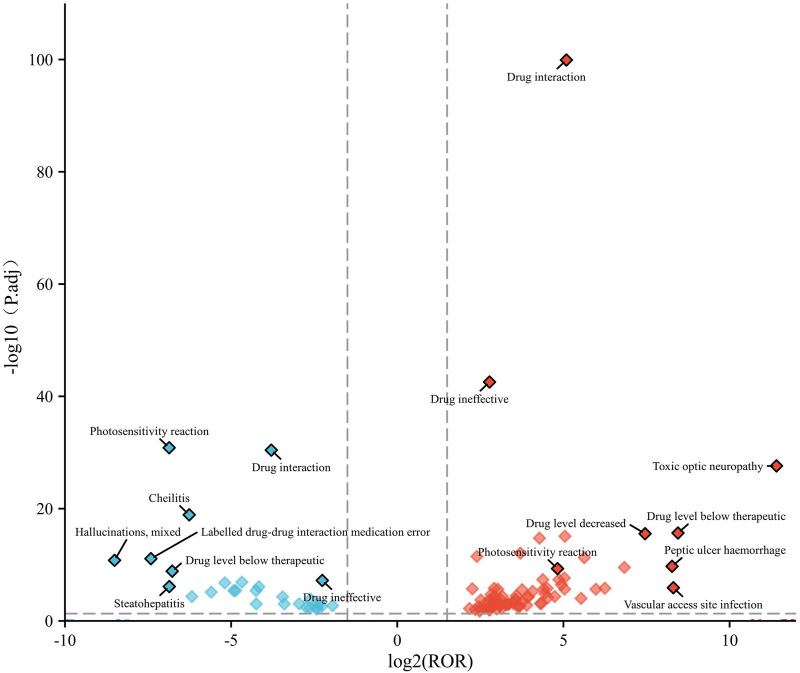
Volcano plot of age-differentiated risk signals for voriconazole in patients with a hematological malignant tumor. *Note:* Horizontal coordinates indicate log2 ROR (left: children; right: adult) and vertical coordinates indicate −log10-transformed adjusted p-values. Significant signals are highlighted in color. p-values are adjusted using the false discovery rate.

We created a volcano plot to visualize the differences in positive PTs between children and adults (Figure 4). The *x*-axis of the volcano plot showed the magnitude of ROR values (log2), the *y*-axis height represented statistical significance, and higher points indicated lower p-values (which are more statistically significant results). Each point in the graph represents a relevant PT.

In the children group, the main terms were “hallucinations, mixed”, “photosensitivity reaction”, “drug interaction”, “labelled drug–drug interaction medication error”, “drug level below therapeutic”, “steatohepatitis”, and “cheilitis”. In the adult group, the main terms were “toxic optic neuropathy”, “drug interaction”, “drug ineffective”, “drug level below therapeutic”, “vascular access site infection”, “peptic ulcer haemorrhage”, and “drug level decreased”.

### 3.4 Time-of-onset of the ADEs

**FIGURE 5:**
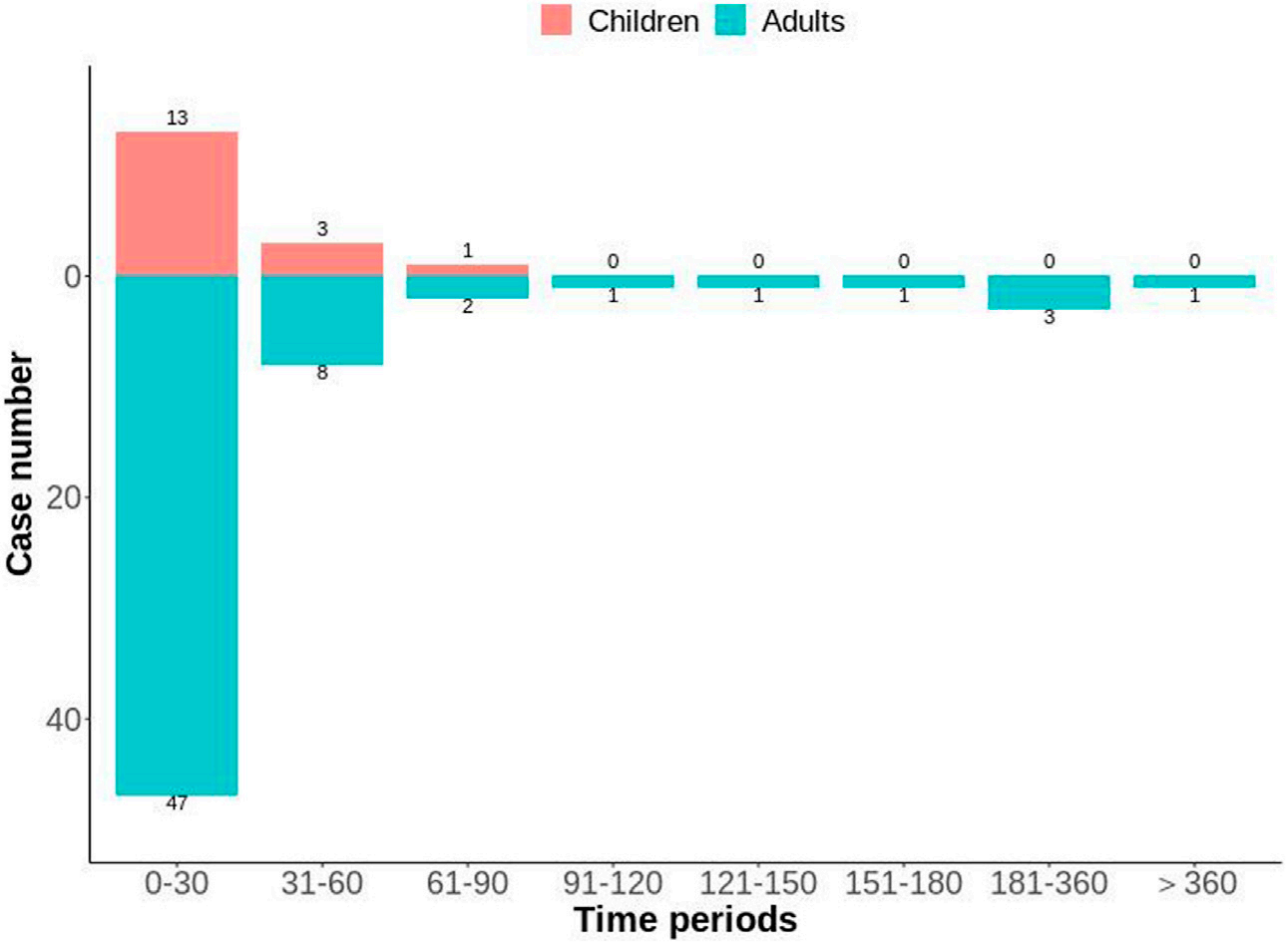
Distribution of time-to-onset of voriconazole-associated adverse reactions in child and adult patients with a hematological malignant tumor.

After excluding reports of missing or inaccurate start/onset date, 81 ADEs were collected, with most cases occurring within 0 and 30 days (n = 60, 74.07%), followed by 31–60 days (n = 11, 13.58%). The number of different time periods in children and adults is shown in Figure 5. The median time-of-onset of the ADE in children and adults was 11 and 8.5 days, respectively.

## 4 Discussion

Voriconazole underwent rigorous pre-marketing clinical trials to ensure its efficacy and safety in treating IFIs. However, the diversity and complexity of the patient population in actual clinical use suggest that there may be ADEs that have not been identified or evaluated fully. We conducted in-depth signal mining and evaluation to explore differences in ADEs between children and adults treated with voriconazole in patients with a hematological malignancy using FAERS. We aimed to provide important information for future clinical use.

Figure 2 showed an upward trend in the number of ADEs related to voriconazole from 2004 to 2020. This trend may have been related to the increased frequency of voriconazole use and increased awareness of ADEs. In particular, the number of reports peaked in 2020, which may have been associated with the coronavirus disease-2019 pandemic (Salmanton-García et al., 2024; Hlaing et al., 2023; Papakonstantinou et al., 2021). Since 2021, there has been a downward trend, which may be related to the development of individualized use of voriconazole in clinical practice.

At the SOC level, commonalities and differences were presented between child and adult groups. The only positive signal that satisfied all four algorithms simultaneously in children was “psychiatric disorders” (Table 2) whereas, in adults, the positive signals were “endocrine disorders” and “hepatobiliary disorders” (Table 3). The percentages of SOC that were significantly higher in children than in adults were “skin and subcutaneous tissue disorders”, “gastrointestinal disorders”, “psychiatric disorders”, and “musculoskeletal and connective tissue disorders” (Figure 3). The percentages of SOC that were significantly higher in adults than in children were “investigations”, “respiratory, thoracic and mediastinal disorders”, “cardiac disorders”, “eye disorders”, and “neoplasms benign, malignant and unspecified (including cysts and polyps)”. In addition, two SOCs were unique to the children group (“ear and labyrinth disorders” and “product issues”), four SOCs were unique to the adult group (“reproductive system and breast disorders”, “congenital, familial and genetic disorders”, “surgical and medical procedures”, and “pregnancy, puerperium and perinatal conditions”). This phenomenon may have been due to the unique physiological state and drug-metabolism characteristics of children (children show greater systemic metabolism of voriconazole than adults) (Leroux et al., 2021).

At the PT level, “photosensitivity reactions” is a more common dermatological complication of voriconazole than in other azole antifungal agents (Malani and Aronoff, 2008). We found that the ROR signal intensity of this PT was higher in children and significant compared with that in adults (Figure 4). This finding provides additional evidence of the need for caution when prescribing voriconazole in children.

In the children group, “hallucinations, mixed” was the PT signal with the highest ROR signal intensity (Table 4). In an observational study of 72 patients aged 14–76 years treated with voriconazole, hallucinations occurred in 12 cases (16.67%). Half of these patients did not report their hallucinations spontaneously. They showed reluctance to describe them, possibly due to embarrassment and other contributing factors (Zonios et al., 2008). A case report and literature review of voriconazole-induced hallucinations and visual disturbances reported 42 cases, three of whom were children (Zheng et al., 2021). In a recent study, a search of multiple databases on drug-induced musical hallucinations identified 27 cases and 21 triggering drugs. Among them, three patients (11.11%) had musical hallucinations induced by voriconazole (Bakewell et al., 2024; Zonios et al., 2008). Voriconazole treatment-related hallucinations may be overlooked by physicians. There are few reports of hallucinations associated with voriconazole in children. However, given the high ROR in the current study, we suggest that whether children experience hallucinations deserves more clinical attention.

In the adult group, “toxic optic neuropathy” had an unusually high ROR signal intensity and high statistical significance (Figure 4). One study revealed six cases of toxic optic neuropathy induced by voriconazole in pharmacovigilance databases (e.g., VigiAcess), and voriconazole was the only drug suspected in two cases (Orssaud et al., 2021). Mounier et al. reported a case of ophthalmic complications possibly caused by toxic optic neuropathy (Mounier et al., 2018). Understanding the mechanism leading to this neuro-ophthalmic adverse effect is crucial for clinical practice. One meta-analysis indicated a trough concentration >3.0 mg/L to be associated with an increased risk of moderate-to-severe hepatotoxicity, and >4.0 mg/L to be associated with an increased risk of neurotoxicity (Jin et al., 2016). Those data suggest a need for close monitoring of the therapeutic concentrations of voriconazole during treatment. Notably, there were “drug interactions”, “drug level below therapeutic”, and “drug level increased” in children and adults in our study. Studies have shown interactions between voriconazole and carbamazepine, cyclophosphamide, aprepitant, tacrolimus, and letermovir, which are related to the induction or inhibition of metabolic enzymes such as CYP2C19, 3A4, 3a5-2D6. Multiple guidelines recommend monitoring the drug concentration during voriconazole treatment to improve safety and efficacy, preferably with prospective dose optimization based on genotype.

We identified new PTs that were not previously listed in the drug label, such as “generalised tonic–clonic seizures” (Table 5) and “disorientation” (Table 4). This finding: (i) suggests that certain patient groups may be at risk; (ii) demonstrates the importance of ongoing post-marketing surveillance and signal mining for ADEs.

Analyses of time-to-onset of the ADE showed that most ADEs occurred within 0 days and 30 days of dosing (Figure 5). The median time-to-onset of the ADE was 11 days in the children group and 8.5 days in the adult group. These data suggested that close monitoring should be carried out during the initial stage of voriconazole treatment. However, one case occurred in the adult group after 1 year of treatment with voriconazole.

Our study had two main limitations. First, FAERS faces inherent challenges, including incomplete, inaccurate, inconsistent, and delayed reporting of ADEs. These factors may have affected the relevance and accuracy of our results. Second, our analysis was affected by the uneven distribution of cases in FAERS, with more adult patients but a significantly smaller number of children. This uneven distribution of cases may have introduced a bias and limited the applicability of our findings. Further prospective clinical studies are needed to overcome these limitations and provide more reliable insights.

## 5 Conclusion

We used four algorithms (ROR, PRR, BCPNN, MGPS) to mine the signals of voriconazole in patients with a hematological malignant tumor. We found similarities and differences in SOC/PT signals between children and adults, but also identified some new PT signals not included in the drug label. In the future clinical use, differentiated pharmaceutical monitoring should be carried out for children and adults, and personalized dosing measures, such as therapeutic drug monitoring, should be combined to optimize the dosage, in order to improve the safety of voriconazole in patients with hematological malignancies.

## Data Availability

The raw data supporting the conclusions of this article will be made available by the authors, without undue reservation.
