# Supplementary material for: Data mining and safety analysis of voriconazole in patients with a hematological malignant tumor based on the FAERS database: differences between children and adults
Source: Front Pharmacol. 2025 Jan 24;16:1524702. doi: 10.3389/fphar.2025.1524702 (PMC11802493; doi:10.3389/fphar.2025.1524702)
Supplement: Supplementary file 1 [file Table1.docx]

**Table 1** Major algorithms used for signal detection in this study.

| **Algorithm** | **Equation** | **Criteria** |
| --- | --- | --- |
| ROR | $ROR=\frac{a}{c}\times\frac{b}{d}$  $95\%CI=e^{ln(ROR)\pm1.96\sqrt{\frac{1}{a}+\frac{1}{b}+\frac{1}{c}+\frac{1}{d}}}$ | $95\%CI>1$  $a\geq3$ |
| PRR | $PRR=\frac{a}{(a+b)}\div\frac{c}{(c+d)}$  ${}^{2}=\frac{{(ad-bc)}^{2}\times(a+b+c+d)}{(a+b)(c+d)(a+c)(d+b)}$  $95\%CI=e^{\ln(PRR)\pm1.96\sqrt{\frac{1}{a}-\frac{1}{a+b}+\frac{1}{c}-\frac{1}{c+d}}}$ | $PRR\geq2$  ${}^{2}\geq4, a\geq3$ |
| BCPNN | $V\left( IC \right)=\frac{1}{{(ln)}^{2}}\left[ \frac{b+c+d+\gamma-1}{(a+1)(1+a+b+c+d+\gamma}+\frac{c+d+1}{\left( a+b+1 \right)(a+b+c+d+3)}+\frac{b+d+1}{(a+c+1)(a+b+c+d+3)} \right]$  $\gamma=\frac{{(a+b+c+d+2)}^{2}}{(a+b+1)(a+c+1)}$  $E\left( IC \right)=\log_{2} \frac{(a+1){(a+b+c+d+2)}^{2}}{(a+b+c+d+\gamma)(a+b+1)(a+c+1)}$  $IC025=E\left( IC \right)-2\sqrt{V(IC)}$  $95\%CI=e^{ln(IC)\pm1.96\sqrt{\frac{1}{a}+\frac{1}{b}+\frac{1}{c}+\frac{1}{d}}}$ | $IC025>0$ |
| MGPS | $EBGM=\frac{a(a+b+c+d)}{(a+c)(a+b)}$  $EBGM05=e^{\ln\left( EBGM \right)-1.96\sqrt{\frac{1}{a}+\frac{1}{b}+\frac{1}{c}+\frac{1}{d}}}$  $95\%CI=e^{ln(EBGM)\pm1.96\sqrt{\frac{1}{a}+\frac{1}{b}+\frac{1}{c}+\frac{1}{d}}}$ | $EBGM05\geq2$  $N>0$ |

Abbreviations: ROR: Reporting odds ratio; PRR: proportional reporting ratio; BCPNN: Bayesian confidence propagation neural network; MGPS: multi-item gamma passion shrinker; IC: information component; EBGM: empirical Bayes geometric mean; a: number of reports arising from the suspect adverse event (ADE) and suspect drug; b: number of reports arising from the suspect AE and all other drugs; c: number of reports arising from the suspect drug and other AEs; d: number of reports arising from other drugs and other AEs; CI: confidence interval; χ^2^: chi-squared; IC025: lower limit of 95% two-sided CI of the IC; EBGM05: lower limit of 95% one-sided CI of EBGM.
